# Emerging and re-emerging infectious diseases: challenges and opportunities for militaries

**DOI:** 10.1186/2054-9369-1-21

**Published:** 2014-09-24

**Authors:** Zheng Jie Marc Ho, Yi Fu Jeff Hwang, Jian Ming Vernon Lee

**Affiliations:** Biodefence Centre, Headquarters of the Medical Corps, Singapore Armed Forces, 701 Transit Road, #04-01, Singapore, 778910 Singapore

**Keywords:** Communicable diseases, Emerging, Infectious disease medicine, Military personnel

## Abstract

The communal nature of living and training environments, alongside suboptimal hygiene and stressors in the field, place military personnel at higher risk of contracting emerging infectious diseases. Some of these diseases spread quickly within ranks resulting in large outbreaks, and personnel deployed are also often immunologically naïve to otherwise uncommonly-encountered pathogens. Furthermore, the chance of weaponised biological agents being used in conventional warfare or otherwise remains a very real, albeit often veiled, threat. However, such challenges also provide opportunities for the advancement of preventive and therapeutic military medicine, some of which have been later adopted in civilian settings. Some of these include improved surveillance, new vaccines and drugs, better public health interventions and inter-agency co-operations. The legacy of successes in dealing with infectious diseases is a reminder of the importance in sustaining efforts aimed at ensuring a safer environment for both military and the community at large.

## Introduction

Emerging and re-emerging infectious diseases are threats that military organisations have to guard against, as they cause substantial impact to operations and training. These diseases may arise from within the military community, as spill-over from the surrounding civilian populace, or during military operations and deployments. Biological warfare and bioterrorism are additional possibilities that militaries need to be prepared for.

Since historical times, emerging infectious diseases have impacted militaries, from the Plague of Athens in 430 BC during the Peloponnesian War linked to the poisoning of water reservoirs by the Spartans [1], and the similar Antonine Plague in 166 AD brought back by returning Roman soldiers from the Parthian War [2, 3]. The Thirty Years War from 1618 to 1648 also saw the devastating effects of Typhus (alongside Plague and accompanying starvation) which resulted in 10 million deaths [4], overshadowing 350,000 combat deaths. The weaponisation of naturally occurring diseases, the threat wrought by travelling soldiers returning home with novel diseases, and the disproportionate impact of disease and non-battle injuries (DNBIs) are still relevant to militaries of today. In more recent times, there have been outbreaks reported among military personnel in both peacetime and field deployments. These include a peacetime Salmonella outbreak in a military establishment in India in 2011 due to possible contamination of food [5], the 2013 scabies outbreak among the Queen’s Guards in the United Kingdom after a military exercise in Germany [6], and travellers’ diarrhoea among U.S. military troops deployed during a training and humanitarian mission in El Salvador in 2011 [7].

Deployment of militaries to foreign grounds also exposes troops to local endemic diseases. A classic example of this is malaria. During World War II and the Vietnam War, 124,109 and 24,606 respective cases among U.S. military personnel deployed to the Southwest Pacific and Vietnam were reported [8, 9]. Such infectious diseases have been shown to impact militaries significantly, not only in mortality and morbidity, but also in operational readiness. Even training during peacetime is affected by disease outbreaks, sometimes resulting in the unexpected suspension of military operations [10].

**Figure 1:**
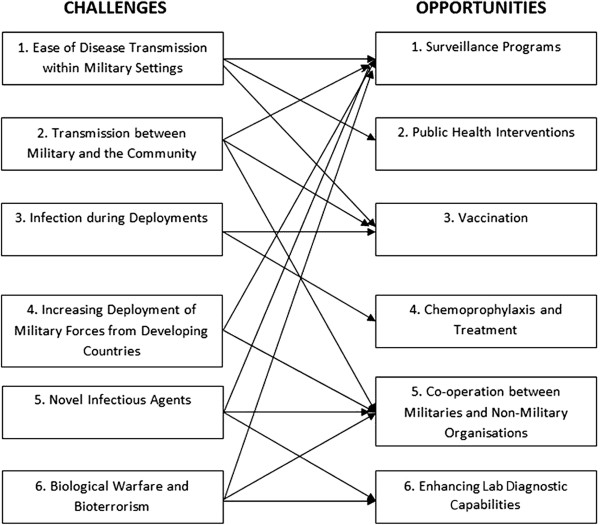
**Challenges and opportunities of emerging and re-emerging infectious diseases in the military.**

Unlike civilian settings, the military may acquire emerging and re-emerging infectious diseases from a myriad of origins and these often spread more readily, posing unique challenges to their prevention and control. At the same time, the frequency and extent of outbreaks in military settings provide opportunities to understand these diseases, and to develop new strategies (Figure 1). This review discusses some of the challenges militaries face from infectious diseases, as well as the societal benefits that militaries have brought to the world as these diseases are managed.

## Challenges in the military environment

### Ease of disease transmission within military settings

Many diseases, especially airborne, food and water borne, as well as vector borne diseases have been shown to spread readily in the military due to the close communal living and training quarters, operational constraints, and unique field hygiene conditions. Harsh environments, physical exertion and sleep deprivation, inadequate hygiene and sanitation, and psychological stress all result in physiological and immunological changes [11]. Although militaries comprise mostly young adults who are physically and mentally fitter, the above factors place them at unusually higher risks of infection.

As a result, there have been numerous reports of infectious disease outbreaks in military settings. These include, but are not limited to, gastroenteritis (particularly Norwalk-like/norovirus outbreaks) [12–14], acute respiratory infections (including influenza) [15–17] and conjunctivitis (particularly due to adenovirus) [18–20]. One report documented that US military trainees staying in a 60 person barracks were more susceptible to contracting acute respiratory infections than those in 8 person rooms, and that the risk was higher during the first few weeks of training before declining to baseline [21]. Similarly, ease of transmission due to close living quarters resulted in an Influenza A(H1N1) pdm09 outbreak in a Swiss military boot camp in 2010 involving 105 of 750 recruits [16]. These incidences reflect the need for preventive measures to protect against future incidences.

### Transmission between military and the community

Some outbreaks in military settings are linked to an increased incidence of disease in the local civilian population. Although the extent of interactions between military and civilian elements vary across militaries, some mixing is inevitable either during transit of personnel from one setting to another, or through socio-civic duties such as disaster relief or community programs.

Amongst the many examples, a Hepatitis A outbreak from 1976 to 1977 in a U.S. military was linked to a nearby childcare facility [22], and concurrent civilian and military outbreaks of Human Adenovirus type 14 in Texas and Oregon in 2007 had possible civilian epidemiological links [23]. There were also reports of concurrent outbreaks of disease in both community and military settings during the outbreaks of enteroviral conjunctivitis in Singapore in 2005 [24] and mumps in Luxembourg in 2008 [25]. Significantly, both of these outbreaks occurred in small countries with military camps in close proximity to the population where increased opportunities for mixing occur.

### Infection during deployments

**Figure 2:**
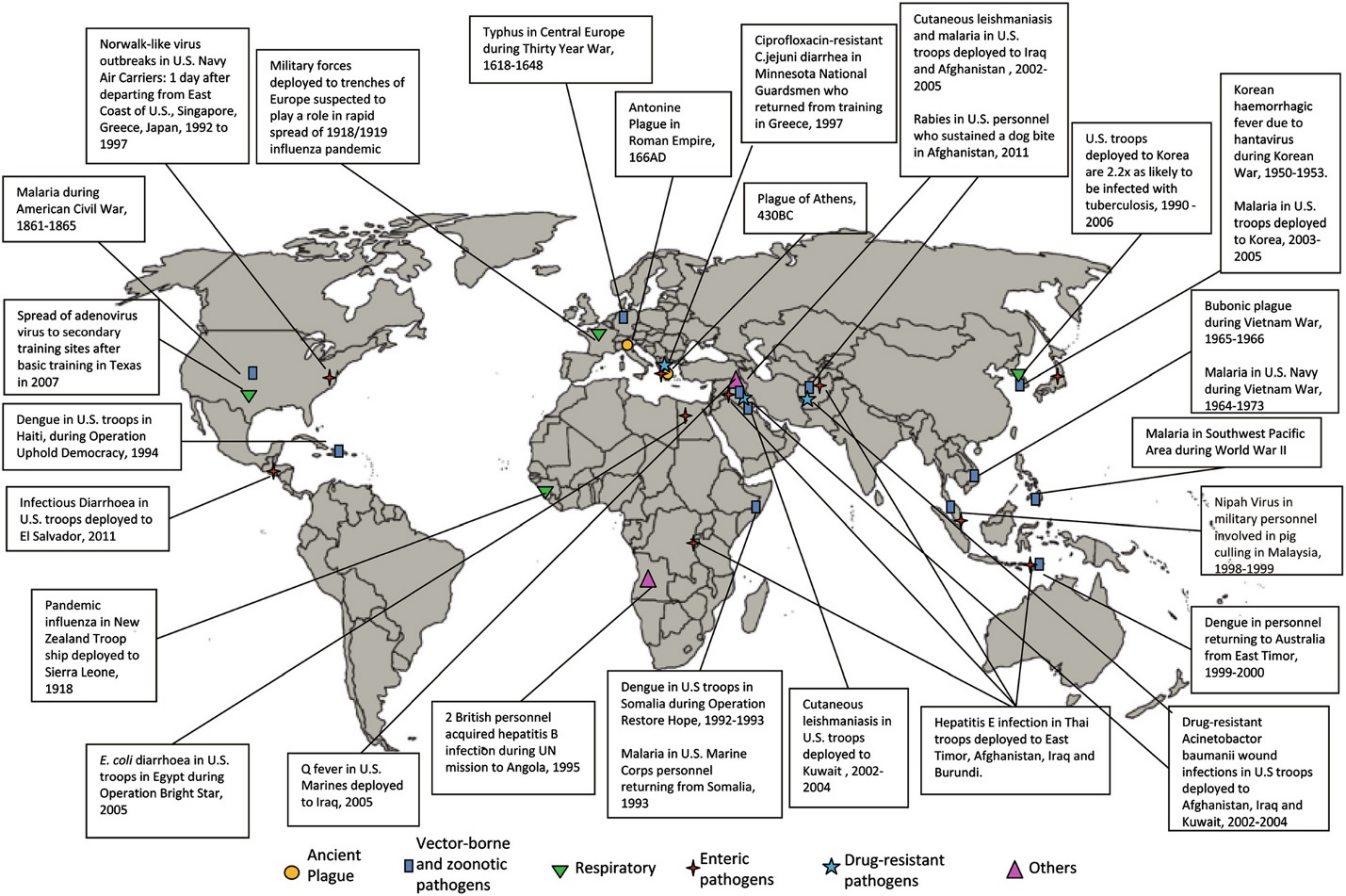
**Selected infectious diseases during military deployments** **[** [1] **-** [4] **,** [7] **-** [9] **,** [15] **,** [26] **,** [28] **-** [30] **,** [39, 41] **,** [44] **,** [50] **-** [61] **].**

Military deployments are commonplace in this age of globalisation and international co-operation, and this coincides with the fact that infectious diseases cross borders more frequently than ever before. Field and rural deployments place personnel at risk of new pathogens not normally encountered. As early as the First World War, the return of military forces from deployments to the trenches of Europe was suspected to have played a role in the rapid spread of the 1918/19 influenza pandemic [26]. More recently, 11 gastroenteritis outbreaks (10 caused by viruses) among British troops deployed overseas from 2002 to 2007 were documented [27]. Personnel returning to Australia from East Timor were also later diagnosed as having been infected with dengue from 1999 to 2000 [28], as did US servicemen infected with the malaria parasite in Iraq, Afghanistan and Korea from 2003 to 2005 [29]. Table 1 shows a selection of infectious diseases during more recent major military deployments and measures implemented (See also Figure 2). Many of these were ground breaking for their time, and provided evidence for their routine use today.

**Table 1:** **Infectious diseases during military deployments and measures implemented**

Operation	Year	Infectious diseases	Measures implemented
**American Civil War**	1861 to 1865	Malaria [30]	Use of Quinine
**World War I**	1914 to 1918	1918 Influenza [31]	Improvements in respiratory hygiene and isolation
		Trench Foot [32]	Footwear modifications
			Foot protection (grease, borated talc and camphor)
			Measures to improve trench and boot drainage
		Tetanus [33]	Prophylactic Anti-Tetanus serum to wounded
**World War II**	1939 to 1945	Wound infections [34, 35]	Use of Dakin’s solution for antisepsis
			Use of Sulfanilamide and Penicillin
		Scrub Typhus [36]	Development of delousing strategies
		Malaria [37]	Use of Atabrine
		Lymphatic Filariasis [38]	
**Korean War**	1950 to 1953	“Korean Haemorrhagic Fever” (Hanta virus) [39]	Improvement in environmental health measures
**Vietnam War**	1953 to 1975	Malaria and Dengue [40]	Mosquito nets and repellents, Antimalarials including Mefloquine - designed by army to counter Chloroquine resistance.
		Bubonic Plague (Yersinia pestis) [41]	Flea insecticide and repellents
			Immunisation with plague vaccines
			Protective clothing and Rat proofed dwellings
**Persian Gulf War**	1990 to 1991	Preparedness against Biological Warfare [42]	Anthrax, Botulinum, Meningococcus vaccines and Hepatitis A immunoglobulins
**Operation Restore Hope**	1993	Malaria [43]	Use of Mefloquine and Doxycycline
**Operation Enduring**	2001 to 2011	Leishmaniasis [44]	Genus specific probe for diagnosis
**Freedom, Operation IraqiFreedom, Operation New**			Treatment modalities under investigational new drug protocols
**Dawn**			Better shelters and insect repellents
		Malaria [45]	Use of rapid diagnostic assays and Tafenoquine
		Norovirus and Shigella [46–48]	Use of rapid diagnostic assays
			Segregation and enforcement of hygiene
		Multidrug Resistant Wound infections and Nosocomial Transmission (especially Acinetobacter baumanii) [49]	Improvements in infection control practices, antibiotic restriction policies
			Admission surveillance cultures of wounded soldiers and contact isolation
			Need for new antibiotics targeting resistant Gram negative bacteria

The French Armed Forces reported that from 1999 to 2009, the rate of food borne disease outbreaks was significantly higher in servicemen deployed overseas (2.4 outbreaks/100,000 in France vs. 26.7 outbreaks/100,000 overseas). These were attributed to a lack of hygiene in operations and the consumption of unsafe local food [62]. Likewise, more than one-half of the 865 infectious diarrhoeal outbreaks in the Israeli military from 1988 to 2002 were in field units [63]. Diseases also tend to spread more efficiently during deployments, as seen by the development of four outbreaks of Norwalk-like viruses on board US Navy aircraft carriers from 1992 to 1997 which were attributed to crowding [50].

Military personnel are often also involved in disaster management, where they interact with displaced populations, themselves at risk of infectious diseases [64]. Water storage or stagnant water around temporary dwellings promotes breeding of vectors, made worse by the absence or interruption of vector control programmes. Furthermore, local food and water safety is poorly enforced, as is the spread of respiratory pathogens when military personnel interact with the local population. Vaccinations, chemoprophylaxis and personal hygiene measures are therefore critical in ensuring force protection when militaries deploy to these areas.

### Increasing deployment of military forces from developing countries

Even developing country militaries that may not possess sophisticated public health capabilities are able to utilise many of the low cost disease prevention measures aforementioned. Some of these forces also double-up as health services for civilians in remote areas, reporting surveillance data to local health authorities, and contributing to national infectious disease surveillance [26]. Militaries also provide surge capacity during natural or man-made disasters when civilian resources are overwhelmed. There has also been an increase in number of developing countries contributing troops for multinational operations [26], and common standards of disease control should be applied to multilateral forces for collective protection. This may entail the distribution of resources from higher-income countries.

### Novel infectious agents

Novel infectious agents such as zoonotic transmission of avian influenza viruses and Middle East respiratory syndrome coronavirus (MERS-CoV) pose challenges in early detection and prevention strategies, including military personnel who reside in or are deployed to affected areas. Returning travellers from affected areas also pose a threat and are difficult to screen if they visit these areas during personal leave. The impact of novel agents is shown by the effect of the 2009 pandemic H1N1 influenza on militaries from overseas importation or through the surrounding general population, with subsequent rapid spread in the closed living environments [65]. In addition, Dual Use Research of Concern (DURC) raises issues with regards to biosafety and biosecurity, such as that which involves the creation of laboratory-modified H5N1 viruses capable of respiratory transmission [66].

### Biological warfare and bioterrorism

Biological agents have been used as weapons of war as early as 600 B.C., when cadavers and animal carcasses were used to weaken the enemy [67]. Blankets from smallpox patients were distributed by the British to Native Americans in 1763, and experiments involving various pathogens (including anthrax and meningococcus) were conducted by the Japanese biological warfare research program during World War II, the latter leading to numerous deaths [68]. Furthermore, biological agents can be utilised through unconventional means. The intentional contamination of salad bars in Oregon with *Salmonella* Typhimurium by the Rajneeshee cult in 1984 [69], the failed attempts to disperse botulinum toxin by the Japanese cult Aum Shinrikyo at various sites in Japan in the 1990s [70], and release of anthrax through mailed letters and packages in the U.S. in 2001 [71] are constant reminders of the ever present yet often concealed threat.

The need to protect against bioterrorism has thus emerged as a priority for most militaries. As bioterrorism can occur from the use of simple naturally occurring diseases to the development or theft of dangerous pathogens, it is important for militaries to prepare for and protect themselves against these threats. This includes the development of good diagnostic facilities for routine infectious diseases detection that also have the capability for detection of novel and dangerous pathogens. Laboratories (both military and civilian) should thus ensure the veracity of biosafety and biosecurity practices to prevent the occurrence of accidents or deliberate releases. This requires collaborations at the international level to strengthen global biosafety and biosecurity initiatives, and investments in capacity building at the local level, especially in settings of lower-resources.

## Opportunities for militaries

Amidst these challenges, there are also multiple opportunities afforded for advancements in the prevention and control against infectious diseases in military settings, and history has shown how military medicine has risen to previous challenges.

### Surveillance programs

Cross border spread and the emergence of novel pathogens have placed surveillance programs, such as that of the French Military Influenza Surveillance System (MISS) and the U.S. Armed Forces Health Surveillance Centre Global Emerging Infectious Surveillance and Response System (AFHSC-GEIS), as centrepieces in the early detection and warning of infectious diseases for the prompt implementation of mitigation and control measures.

These surveillance systems are important for the global surveillance of infectious diseases. Through the US Naval Health Research Centre’s surveillance program, Influenza A(H1N1)pdm09 in two California children was first identified, leading to the promulgation of an alert by the Centers for Disease Control and Prevention (CDC) [72]. This subsequently led to notification of another outbreak among New York students who had travelled to Mexico, and thereafter the detection of the same pathogen in outbreaks in Mexico [72]. These represented sentinel events leading to global awareness of the rapidly evolving situation.

Influenza surveillance can also be found in other armed forces, including the French who have participated in national surveillance through MISS since 1997. Its main objectives are the early detection of influenza outbreaks and monitoring of circulating viral strains [73]. Compared with national estimates, the MISS estimated lower amplitudes of influenza outbreaks in the military from 2008 to 2012, with possible reasons including younger ages, healthier personnel and compulsory influenza vaccination. In the Italian military, diseases such as measles and varicella are notifiable under its surveillance program [74]. These results have been used to guide organisational vaccination policies. For example, after a tenfold increase in measles and fourfold increase in rubella from 1976–1980 to 1991–1995, mandatory MMR vaccination was instituted. Likewise, on noting that influenza was the foremost cause for febrile respiratory illnesses leading to substantial morbidity [75], the Singapore Armed Forces thereafter instituted annual influenza vaccination with significant success [76].

Military surveillance is often a component of wider population-wide protection measures, such as the Medical Surveillance System implemented in support of the United Nations Mission in Haiti [77]. The Peruvian Navy also developed an electronic disease surveillance to rapidly consolidate reportable diseases, thereby allowing earlier detection and control of disease outbreaks in the country. Finally, the Royal Thai Army instituted HIV screening on conscription, thereby serving as an indirect monitor of the national HIV situation [26, 78].

### Public health interventions

Public health interventions are equally as important in the management of infectious diseases in the military. These include contact precautions such as the disinfection of common living surfaces and hand hygiene, and droplet and airborne protection including isolating ill individuals and basic respiratory hygiene education. Food and water hygiene is also important due to the large numbers of individuals sharing common food and water sources. Some of these are more easily enforced in the controlled environments of military bases. However, once in the field, they pose a unique challenge where inadequate hygiene and sanitisation are often lacking. Even so, through innovations such as ready-to-eat meals and a host of water purification methods, militaries attempt to reduce these risks.

Positive examples of successful public health measures include the early identification and isolation of patients during the 1918 influenza pandemic which lowered attack rates in closed settings [79], and enhanced public health measures put in place for critical operations units in the Singapore military during the Influenza A(H1N1)pdm09 pandemic (including symptom monitoring and segregation into smaller subgroups when working) resulting in lower seroconversion rates (17% vs. 44% for other units) [80]. Likewise, a study involving conjunctivitis outbreaks in a military camp showed that for each day to which control measures were delayed, the outbreak itself was extended by 0.82 days [81].

Health education also plays an important role amongst military personnel. This includes basic concepts such as disease recognition and prevention strategies, food hygiene and handwashing, respiratory hygiene, cleaning of common areas, and monitoring for disease symptoms. In addition, select personnel can be further trained in the use of personal protective equipment which range from simple gloves and masks during cleaning of soilages, to full suits against biological attacks.

vAnother area of interest is the use of public health measures for the prevention and containment of infection, especially regarding resistant bacteria in the combat wounded [82]. Environmental studies in military hospitals indicate the presence of nosocomial transmission of multi-drug resistant pathogens and/or existing colonisation prior to injury [83]. These led to the step-up of education pre-deployment, standardisation and enhancement of infection control measures, improved electronic support, and admission screening for resistant bacteria colonisation [49]. In a separate study, the conduct of a 5-day infection control course also showed a 21% improvement in knowledge [84].

Further epidemiological, public health and translational research provide opportunities for future advancements and better interventions to prevent further spread and quick containment of infectious diseases.

### Vaccination

Since the time George Washington ordered the Continental army recruits to be vaccinated with smallpox vaccines in 1777 in response to outbreaks of variola, vaccination has evolved to become an integral part of troop protection against infections [85]. The military has also contributed to the development of many of these vaccines. For instance, the first typhoid vaccine produced for national supply was by the US Army Medical School in 1909, based on British and German production methods. The first inactivated influenza and adenovirus vaccines [86] were also developed by the US military, becoming the first iterations of the vaccines used today. Furthermore, military sponsored research contributed to the eventual licensure of ten vaccines from 1945 to 1955 [87]. Table 2 summarises the role to which militaries have played in the development of vaccines_._

**Table 2:** **Vaccinations and military contributions** [85–89]

Vaccine	Year initiated	Military role
**Smallpox**	1777	Used by the Continental Army in 1777; Used by Prussian Army during the Franco-Prussian War in 1870
**Yellow fever**	1900	Demonstration of etiological agent and vector
**Typhoid fever**	1909	British Army used early forms during the Anglo-Boer War; US Military developed killed typhoid vaccine for US Army and Navy personnel
**Tetanus**	1940	Used by the US Army and Navy from 1940; Used by the Luftwaffe in World War II
**Cholera**	1940, 1980s	Injectable whole cell vaccines given to alert US Military Units until 1973
**Hepatitis A**	1945, 1985	US Military developed immunoglobulins and used in Korea and Vietnam in the 1960s; US Military supported safety, immunogenicity and efficacy studies
**Pneumococcus**	1945	US Military tested first multivalent polysaccharide vaccine
**Diphtheria**	1950s	US Military sponsored reduced dose formulation
**Anthrax**	1950s	Early anthrax vaccines were developed by Dr George Wright of the U.S. Chemical Corps and his colleagues, and were licensed in 1970.
**Adenovirus**	1952-1969	US Military developed killed bivalent and oral attenuated vaccine
**Influenza**	1957	US Military developed first iterations of influenza vaccines and conducted trials among US service members
**Japanese Encephalitis**	1950s, 1980s	US Military performed vaccination efforts in World War II and later supported inactivated vaccine studies
**Plague**	1960s	US personnel in Vietnam and Southern Vietnam soldiers vaccinated in the 1960s
**Meningococcus**	1968	US Military developed polysaccharide vaccine and supported clinical trials; Adopted by the Israeli Army in 1994
**Rubella**	1972	US Military developed technique for isolating virus used to develop the vaccine
**Measles**	1980	US Military provided funding for development of an attenuated vaccine

Vaccines continue to be under development through military sponsored studies. For instance, a Phase 3 trial on an oral adenovirus Type 4 and 7 vaccine was conducted among US Military recruits [90], with subsequent FDA approval in 2011. Continued research into vaccines against dengue, norovirus and HIV continue to be much anticipated by both military and non-military medical communities.

Militaries across the world have developed their own vaccination programs that are often more comprehensive than their respective civilian populations. The U.S. military has routine immunisation for infectious diseases influenza and adenovirus, and travel immunisation depending on exposure, such as variola vaccination for personnel in areas at higher risk for release of smallpox as a weapon [91]. This being so, a World Health Organisation Survey in the 1990s noted that while 90% of the 52 militaries surveyed claimed to have compulsory military immunisation schedules, compulsory vaccination for different infectious diseases varies across militaries from 19% for Measles, Mumps and Rubella to 87% for tetanus [92].

### Chemoprophylaxis and treatment

The use of chemoprophylaxis and treatment against infections in the military continues to be an extensive field with opportunities for further exploration. During the American Civil War from 1861 to 1865, three studies on antiseptics (bromide, turpentine and nitric acid) showed reductions in mortality from hospital gangrene [93]. Almost 80 years later during the Second World War, the critical discovery of penicillin’s antibiotic effects propelled it to become second highest priority (behind the atomic bomb) by the U.S. War Production Board [94]. Its use has often been cited as pivotal to success of the Allied Forces [95].

Apart from antibiotics, military medicine has also helmed developments and innovations in the use of anti-parasitic and anti-viral chemoprophylaxis. The anti-malarial drug mefloquine was developed by the Walter Reed Army Institute in the 1960s, in collaboration with the World Health Organisation and Hoffman-LaRoche [96]. Further studies also showed synergism between atovaquone and proguanil, with subsequent co-administration trials conducted at various sites by military-associated laboratories in Kenya, Brazil and Indonesia. The Influenza A(H1N1)pdm09 pandemic also saw the effective use of oseltamivir ring prophylaxis in the Singapore Military in localised outbreaks to bring about significant reductions in the reproductive number (the number of new cases attributable to the index case) from 1.91 to 0.11, with significantly reduced rates of infection [97].

Other drugs have been, or are being evaluated for possible benefits of chemoprophylaxis in the military setting. These include the successful use of rifaximin for the prevention of travellers’ diarrhoea among travellers and deployed military personnel [98], and dilute Dakin solution for angioinvasive fungal infection in the combat wounded [99].

Militaries around the world have also implemented chemoprophylaxis programs against specific threats. The U.S. military has guidelines for different infectious diseases including pre-exposure prophylaxis for malaria during deployments to affected areas and post-exposure prophylaxis for anthrax and meningococcus exposure [91]. The Republic of Korea Army instituted a malaria prophylaxis program in 1997, and no malaria deaths have been reported since [100]. In Singapore, malaria prophylaxis is routinely used for travel to malarious locations globally. At the same time, on Singapore’s Tekong Island which houses a military training facility, an integrated combination malaria eradication strategy since 2006 has negated the need for malaria prophylaxis [101].

### Co-operation between militaries and Non-military organisations

Collaborations between different militaries, as well as between militaries and civilian organisations on various levels are important in the prevention and control of infectious disease, and are best performed before the onset of outbreaks or epidemics. On a national level, the interface between the Peruvian military and Royal Thai Army (described earlier) with their respective national health networks allowed for smoother sharing of valuable information, so as to formulate more appropriate responses and measures where necessary [78]. On a larger scale, international networks allow participating countries to pool resources, providing greater equity. For example, the AFHSC-GEIS network picked up 76 outbreaks in 53 countries from 2008 to 2009, and involved supporting civilian entities in 48% of outbreak investigations, including a number of WHO regional reference laboratories [102].

Civil-military collaborations also play an important role during humanitarian efforts. After the earthquake in Haiti in 2010, security established by the military allowed civilian medical personnel more ready access to patients, and also provided critical equipment and supplies [103]. The Oslo Guidelines, updated by United Nations in 2007, provides guidelines for international military aid in disaster relief operations- one key point being that military assets should only be utilised when there are no viable alternatives, and with due regard for the sovereignty and leading role of local authorities [104, 105].

### Enhancing lab diagnostic capabilities

As early detection plays an important role in the mitigation of infectious diseases, militaries have a responsibility to enhance their laboratory capabilities for diagnosis. To maximise the use of resources, militaries should work together with civilian laboratories to strengthen national capabilities in the detection of novel infectious agents under International Health Regulations, exemplified by the early detection of Influenza A(H1N1)pdm09 by a U.S. military laboratory [72]. In addition, militaries sometimes require rapid diagnosis of common diseases especially during field deployments where full laboratory facilities may be unavailable. Enhancing laboratory capacity in militaries should therefore extend to improving capabilities in field and austere conditions where diseases are often encountered.

## Conclusion

Although the ever-changing infectious diseases scene and harsh operational and training environment pose unique and sometimes seemingly daunting challenges to military organisations, it also presents many opportunities for scientific advancements in the areas of prevention, mitigation and control. From surveillance to treatment and public health measures, the field of infectious diseases in the military has, more than ever before, many puzzles waiting to be solved. Military healthcare providers and administrators involved in this worthwhile endeavour should also be encouraged that the work they do goes beyond accruing direct benefits for militaries, often flowing to the greater community at large. As such, continued investment in military surveillance, research and management of infectious diseases is important to ensure that the world we live in is safer from the threat of diseases.

## Authors’ information

All authors are from the Biodefence Centre, Headquarters of the Medical Corps, Singapore Armed Forces. LJM (MBBS, PhD, MPH, MBA) is Head of the Centre, Associate Professor at Saw Swee Hock School of Public Health, National University of Singapore, and Visiting Consultant at the Ministry of Health, Singapore and Communicable Disease Centre, Singapore.
